# A NitroPure Nitrocellulose Membrane-Based Grapevine Virus Sampling Kit: Development and Deployment to Survey Japanese Vineyards and Nurseries

**DOI:** 10.3390/v15102102

**Published:** 2023-10-17

**Authors:** Mizuho Nita, Taylor Jones, Diana McHenry, Elizabeth Bush, Charlotte Oliver, Akira Kawaguchi, Akiko Nita, Miyuki Katori

**Affiliations:** 1 Alson H. Smith Jr. Agricultural Research and Extension Center, Virginia Polytechnic Institute and State University (Virginia Tech), Winchester, VA 22602, USAebush@vt.edu (E.B.); clo130@vt.edu (C.O.);; 2 Department of Law and Economics, Shinshu University, Nagano 390-8621, Japan; 3 National Agriculture and Food Research Organization (NARO), Western Region Agricultural Research Center, Hiroshima 721-8514, Japan

**Keywords:** GLRaV2, GLRaV3, GRBV, NitroPure nitrocellulose, grapevine viruses

## Abstract

We developed a NitroPure Nitrocellulose (NPN) membrane-based method for sampling and storing grapevine sap for grapevine virus detection. We devised an efficient nucleic acid extraction method for the NPN membrane, resulting in 100% amplification success for grapevine leafroll-associated virus 2 (GLRaV2) and 3 (GLRaV3), grapevine rupestris stem pitting-associated virus (GRSPaV), grapevine virus A, grapevine virus B, and grapevine red blotch virus (GRBV). This method also allowed the storage of recoverable nucleic acid for 18 months at room temperature. We created a sampling kit to survey GLRaV2, GLRaV3, and GRBV in Japanese vineyards. We tested the kits in the field in 2018 and then conducted mail-in surveys in 2020–2021. The results showed a substantial prevalence of GLRaV3, with 48.5% of 132 sampled vines being positive. On the other hand, only 3% of samples tested positive for GLRaV2 and none for GRBV.

## 1. Introduction

Among perennial crops worldwide, the grapevine can harbor the largest number of viruses, exceeding 70 [1,2,3]. While most of these viruses are considered minor threats due to their limited economic impact or restricted geographic range, certain viruses, such as the leafroll virus complex and grapevine red blotch virus, pose substantial threats, inflicting severe damage in infected vines [2,3,4,5,6].

The Japanese wine industry boasts a venerable heritage, tracing back to the early 19th century when the vineyard was established in the Yamanashi prefecture in 1875 [7]. However, it was not until the 20th century that wine production in Japan truly gained momentum. Despite the formidable challenges posed by the climatic conditions for grape cultivation, the Japanese wine industry has witnessed a steady and continuous expansion. Presently, the country is home to approximately 300 wineries, primarily concentrated in the Yamanashi, Nagano, and Hokkaido prefectures [7,8]. Although some studies have reported the presence of grapevine viruses [9,10,11,12], there is limited information on the phytosanitary status of vineyards and nurseries regarding grapevine viruses.

Grapevine leafroll disease (GLD) exists across all grapevine-growing regions worldwide [4,13,14,15]. Research conducted in various countries has demonstrated the significant impact of GLD on vine vigor, fruit yield, and grape quality [13,15,16,17,18]. Direct crop losses can range from 10% to 40% in vineyards severely affected by GLD and planted with susceptible cultivars [18]. Furthermore, the infection is commonly associated with uneven ripening of berries, compromised coloring, and diminished sugar levels [4,17,19]. Moreover, the vectors responsible for transmitting these viruses, mealybugs and scale insects, are prevalent in numerous grape-growing regions [20,21,22,23,24].

The viruses involved in GLD are collectively called grapevine leafroll-associated viruses (GLRaVs) and are designated as GLRaV1, 2, 3, 4, 7, and 13 [1,11]. These viruses belong to the family *Closteroviridae*. GLRaV2 is classified in the genus *Closterovirus*. GLRaV7 falls within the genus *Velarivirus.* GLRaV1, 3, and 4 (including strains GLRaV5, 6, 9, De, Pr, and Car [3,25,26]), and 13 are grouped under the genus *Ampelovirus*.

GRBV, a member of the *Geminiviridae* family, is gaining attention among growers due to its adverse effects on yield and quality [27,28,29]. A 42% yield loss was reported in the Cabernet franc vineyard in British Columbia [30]. Wines made using berries from GRBV-infected vines exhibited lower ethanol content due to poor sugar accumulation and elevated pH [31]. GRBV has been identified in various regions across the United States, including California, New York, Virginia, Maryland, Pennsylvania, Texas, and Washington [13,32,33,34].

Recognizing the significant impact of virus diseases in many grapevine-growing regions worldwide, surveys have assessed the presence of viruses in vineyards. Surveys conducted in California, Idaho, Missouri, New York, Oregon, Virginia, and Washington State revealed the occurrence of various viruses, including GLRaV1, 2, 3, 4, 7, and GLRaV4 strains 5, 9, and Car, GVA, GRSPaV, grapevine fanleaf virus (GFLV), grapevine fleck virus (GFkV), Arabis mosaic virus (ArMV), tomato ringspot virus (ToRSV), GRBV, and grapevine vein clearing virus (GVCV) [13,35,36,37,38]. Our investigations have indicated that GLRaV3, GRSPaV, and GRBV are Virginia’s most prevalent grapevine viruses [6,33].

The survey methodologies often start with collecting grapevine tissues during the growing season or winter, followed by nucleic acid extraction and subsequent detection using either ELISA or molecular-based techniques such as PCR, RT-PCR, or real-time PCR [13,39,40,41,42,43]. Due to the similarities of symptom development among different viruses and nutrient deficiencies (e.g., GLD, GRBD, and potassium deficiency are associated with red leaves), techniques such as ELISA, DNA-based, hyperspectral imaging, and mass spectrometry are used for accurate diagnostics [44,45,46,47]. The sampling process typically involves coordinating with growers for site visits, traveling to the vineyards, and collecting petioles from suspected grapevines, which are then placed in sealed containers or bags and stored on ice. Subsequently, the samples are transported to cold storage (at 4 °C to −80 °C) to maintain their viability until nucleic acid extraction.

Alternatively, the utilization of various membranes, such as FTA cards (Flinders Technology Associates), Hybond N^+^ Nylon, and NitroPure Nitrocellulose (NPN) membranes, has been proposed as a more straightforward and robust method for virus sampling and testing. Plant sap is blotted before being utilized in multiple assays such as PCR, RT-PCR, qPCR, and TBIA (tissue blot immunoassay) [48,49,50]. Notably, these membranes can bind nucleic acids and preserve virus integrity at room temperature (20–25 °C) for extended periods [48,49,50]. FTA cards and Hybond N^+^ Nylon membrane spotting techniques have been demonstrated to be effective in detecting grapevine viruses and other pathogens, including but not limited to GLRaV1, 3, 5, 9, GVA, GVB, GRSPaV, GFkV, *Xylella fastidiosa*, and phytoplasmas (e.g., Peach rosette and Western X-disease) [49,50]. Similarly, FTA cards and NPN membranes have proven effective for cucumoviruses and potyviruses [48].

This study examined multiple protocols for membrane-based sampling and testing methods targeting ten grapevine viruses to devise a new NPN membrane-based grapevine virus sampling kit. Leveraging this kit, we surveyed grapevine viruses from 2018 to 2021 in Japan to determine the prevalence of GLRaV2, GLRaV3, and GRBV.

## 2. Materials and Methods

Grape tissue sampling. Fifteen fresh grapevine petiole tissues were sampled from each grapevine. Samples were arbitrarily taken from the canopy’s lower and upper parts to include both young and old petioles. We sampled at least three shoots from each side of the trunk since vines in sampled vineyards were trained bilaterally. This was our attempt to improve the chance of obtaining tissues that may contain grapevine viruses that tend to be unevenly distributed within a vine [51]. Samples were collected from July to September (i.e., ~one month after bloom to harvest). The sampled petioles were placed in Ziploc bags, immediately stored in an insulated cooler box containing ice, and transported to the laboratory. Subsequently, the petiole samples were cut into sections of 0.25 g using sterile razor blades and transferred to grinding bags (BIOREBA, Reinach, Switzerland) containing 5 mL of filter-sterilized extraction buffer (EB) (1.59 g/L Na_2_CO_3_, 2.93 g/L NaHCO_3_, pH 9.6 containing 2% polyvinylpyrrolidone-40, 0.2% bovine serum albumin, and 0.05% Tween 20) [52]. The samples were homogenized using a Homex 6 mechanical grinder (BIOREBA, Switzerland), and the extracts were transferred to 1.5-mL microcentrifuge tubes. These samples were then stored at −80 °C for a maximum of 24 months before the start of this experiment. We used samples from our earlier investigations [6,33]. In addition, we collected 150 samples from known virus-positive vines at two commercial vineyards in Orange County, VA, USA, and at an experimental vineyard in Winchester, Virginia, in 2015.

### 2.1. Development of the NPN Membrane-Based Grapevine Virus Sampling Kit

#### 2.1.1. Validation of the NPN Membrane

The 0.45 μm pore size NPN membrane (Maine Manufacturing LLC, Sanford, ME, USA) has demonstrated success in tissue-blot and RT-PCR assays for cucumoviruses and potyviruses [48]; however, its application to grapevine viruses has not been explored. The FTA classic cards (Whatman, GE Healthcare Bio-Sciences, Pittsburgh, PA, USA) are used in tissue-blot and RT-PCR/qPCR analyses of some grapevine viruses [49]. The FTA card was used in this study to validate the efficacy of the NPN membrane, which is the focus of this work.

Each virus-positive sample was thawed on ice, and 10 μL of the plant extract, prepared with the method described above, was carefully spotted onto both the NPN membranes or FTA cards using a pipette. Subsequently, the membranes or cards were left to air dry for 24 h on the laboratory bench [49]. Once thoroughly dried, 3 mm discs containing the blotted and dried samples were extracted from the membranes or cards using a 3 mm micro-punch (Harris Uni-core Sampling Tool 3.0, Electron Microscopy Sciences, Hatfield, PA, USA).

Each disc from the NPN membranes was added to 200 μL of GES buffer (consisting of 0.1 M glycine, 0.05 M NaCl, 1 mM EDTA, and 0.5% Triton X-100) [49]. The FTA card discs were subjected to two washes with FTA^®^ purification reagent as per the manufacturer’s protocol (Whatman), followed by two washes with TE buffer (10 mM Tris, 0.1 mM EDTA, pH 8.0) [49]. These samples were incubated at 95 °C for 10 min, followed by 30 s of vortexing and placement on ice.

Subsequently, 2 μL aliquots of the suspended nucleic acid from the NPN membrane discs or the dry FTA card discs were added to a total volume of 25 μL, consisting of 13.4 μL nuclease-free water, 2.5 μL 10X PCR buffer (New England Biolabs, Ipswich, MA, USA), 2.5 μL sucrose/cresol red solution (20% *w*/*v* sucrose, 1 mM cresol red) (Sigma-Aldrich Co., LLC, St. Louis, MO, USA), 1.25 μL 20 μM virus-specific forward primer, 1.25 μL 20 μM virus-specific reverse primer, 1.25 μL 100 mM dithiothreitol (Sigma-Aldrich Co., LLC, St. Louis, MO, USA), 0.5 μL 10 mM dNTPs (Invitrogen, Grand Island, NY, USA), 0.1 μL 40 U/μL RNaseOUT (Invitrogen), 0.035 μL 200 U/μL Superscript III RTase (Invitrogen), and 0.25 μL 5 U/μL Taq DNA polymerase (New England Biolabs) [41,49,53]. For conventional PCR reactions (i.e., for GRBV), 0.135 μL of water substituted the RTase or RNaseOUT (Table 1). Water controls and positive controls (i.e., not deposited on a membrane) were included to validate the accuracy of the test results. The PCR products were visualized using gel electrophoresis on a 1.5% agarose gel in TBE (89 mM Tris Borate, 2 mM EDTA, pH 8.2) stained with GelRed (Biotium, Hayward, CA, USA) and examined using a UV trans-illuminator and imager.

#### 2.1.2. Maceration Buffer

Two maceration buffers were evaluated in addition to the water control: an extraction buffer (EB) (as described earlier) and the commercially available Grapevine Sample Buffer (GSB) (#M004-K1, AC Diagnostics, Fayetteville, AR, USA). Each sample comprised five grapevine petioles randomly selected from the infected vine’s canopy. The petiole samples were then cut into small pieces using household scissors. The scissors were disinfected with 10% bleach for 1 min between samples and rinsed with water. Approximately 1 g of petiole tissue was placed at the bottom of a small, sterile disposable medicine cup (cat#EF5639, Daigger Scientific, Vernon Hills, IL, USA), which was sufficient to fill the entire bottom of the cup. Subsequently, 1 mL of EB, GSB, or water was added to each sample cup. Using the blunt ends of sterile 15 cm long wood applicators (cat#72303, Electron Microscopy Sciences, Hatfield, PA, USA), the petiole tissue was gently mashed to break up the tissue and then macerated for 60 to 90 s. The applicator was then dipped into the mix and gently touched against an NPN membrane to blot the solution. Blotting was performed twice per sample. The membrane was left to dry for a minimum of 12 h.

#### 2.1.3. Membrane Preparation for PCR Reactions

Five methods (Methods A, B, C, D, and E in Table 2) were examined to prepare the membrane, which involved washing the membrane in buffers to facilitate nucleic acid recovery. Each method used a 3 mm disc from an NPN membrane containing a blot of either GLRaV3- or GRBV-positive grape sap using a sterile micro-punch. The micro-punch was cleaned by immersing it in 10% household bleach with gentle agitation for 60 s, and then rinsing it with deionized water for another 60 s and blotting it dry between samples.

In Method A, 3 mm membrane discs were placed in a tube used in the subsequent RT-PCR and PCR reactions. Methods B and C followed the NPN membrane protocols used for other plant viruses [48]. The discs were rinsed with either 200 μL of FTA reagent (Method B) or 5% Triton X-100 (2-[4-(2,4,4-trimethylpentan-2-yl)phenoxy]ethanol) (Method C) in deionized water. Triton X-100 is a non-ionic detergent for cleaning debris. The samples were vortexed for 5 s and left at room temperature for 5 min. The liquid was then removed, and the discs were rinsed twice in 200 μL TE buffer and allowed to air dry for at least 24 h before being used in the RT-PCR/PCR reactions for GLRaV3/GRBV.

Methods D and E followed the previously published protocols for FTA cards and Hybond N^+^ Nylon membranes [49]. In method D, 50 μL GES was used; in method E, 1% beta-mercaptoethanol was added to 50 μL GES. Samples were incubated at 95 °C for 10 min, vortexed, and then placed on ice for immediate use in the RT-PCR and PCR reactions.

#### 2.1.4. PCR Templates

Two types of templates were examined for use in the PCR reactions: the 3 mm NPN disc (Methods A, B, C, D1, and E1) or the 2-μL solution containing the disc after vortexing for 90 s (Methods D2 and E2) (Table 2). For each method, 48 samples from GLRaV3-positive vines were tested using a one-step RT-PCR protocol [46] with the GLRaV3 primer set shown in Table 1. We also tested GRBV using 48 GRBV-positive samples using a conventional PCR protocol [29] with the primers specified in Table 1. Water and positive controls (=previously confirmed positive specimens from [6]) were included to validate the test results. The PCR products were visualized using gel electrophoresis, as described previously.

#### 2.1.5. Validation Testing

Twenty-five fresh petiole samples from grapevines known to be infected with GLRaV2, GLRaV3, GRSPaV, GVA, GVB, and GRBV were collected from the field in July 2015. Ten petioles were collected from each vine, and samples were blotted onto the NPN membrane as described above. The tissue was ground in GSB, and nucleic acid recovery was performed using GES with 1% beta-mercaptoethanol (Method E2). The RT-PCR or PCR reaction template was 2 μL of the supernatant. PCR-positive samples were cleaned (QIAquick PCR Purification Kit, QIAGEN, Valencia, CA, USA) and sequenced at the Virginia Bioinformatics Institute, Virginia Tech (Blacksburg, VA, USA) using an ABI 3730 DNA sequencer (Applied Biosystems, Foster City, CA, USA).

#### 2.1.6. Stability of Samples after 18 Months of Storage

The membranes containing several subsamples from these samples were stored at 20–25 °C on the laboratory benchtop in a Rubbermaid container stacked with paper dividers. After 18 months, subsamples from the membranes were tested to confirm the long-term storage viability of recoverable nucleic acid.

### 2.2. The NPN Membrane Kit in the Real World

#### 2.2.1. Grapevine Virus Surveys in Japan

Site visits were conducted in 2018, and our lab members used the kit. Then, due to the COVID-19 pandemic, we were forced to carry out a mail-in survey in 2020–2021. For the survey, we packaged the NPN membrane-based kit for growers. The kit included an NPN membrane with protective papers, wood sticks (~5 mm diameter × 15 cm length), razor blades, GSB buffer, paper towel, and detailed instructions (Table 3). Additionally, we provided a video link (https://youtu.be/Yyes0YPY-Fs) (accessed on 20 September 2023) to assist growers. Fifty kits were distributed to vineyard managers and winery owners. We asked them to collect two samples per kit and included a survey to collect the location, cultivar, and level of symptom expression for each sample.

Upon receiving the samples in the USA, nucleic acids were extracted from the NPN membrane discs using a 50 µL reaction containing 1x GES (0.1 M of glycine, 0.05 M of NaCl, 1 mM of EDTA) and 1% beta-mercaptoethanol and incubated at 95 °C for 10 min [48].

GLRaV2 and GLRaV3 were detected using a multiplex RT-qPCR approach with Bioline’s SensiFAST Probe No-ROX One-Step Kit (BIO-76001) (Table 4). The reaction mixture included 300 nM of GLRaV2 primers, 100 nM of GLRaV2 probe, 700 nM of GLRaV3 primers, 250 nM of GLRaV3 probe, reverse transcriptase, 0.8 U/µL of RNase inhibitor, and 5 mM of dithiothreitol. Two microliters of the released nucleic acids were used in a 20 µL total volume. The amplification cycle was 45 °C for 10 min, 95 °C for 2 min, followed by 40 cycles of 95 °C for 5 s and 60 °C for 30 s. Two technical replicates were employed.

GRBV was detected using Bioline’s SensiFAST Probe No-ROX Kit (BIO-86020) in a multiplex qPCR assay with an internal control (=GRBV-negative grapevine) (Table 4). The reaction mixture contained 900 nM of GRBV primers, 900 nM of COX primers, 250 nM of both probes, and 1.6 µL of the released nucleic acids, totaling 20 µL. The amplification cycle for this assay involved an initial step of 95 °C for 5 min, followed by 40 cycles of 95 °C for 10 s and 59 °C for 50 s. Two technical replicates were included in the analysis.

#### 2.2.2. Genetic Variability of GLRaV3 Samples in Japan

RT-PCR was performed using Invitrogen’s SuperScript III One-Step RT-PCR System (Table 2). The reaction mixture consisted of 500 nM of primers, 0.8 U/µL of RNase inhibitor, 5 mM of dithiothreitol, and 2 µL of the released nucleic acids, with a total volume of 25 µL. The thermal profile for the amplification included initial steps of 52 °C for 30 min and 94 °C for 2 min, followed by 35 cycles of 94 °C for 30 s, 54 °C for 45 s, and 68 °C for 1 min, with a final extension at 68 °C for 2 min. The resulting PCR products were purified using the QIAGEN QIAquick PCR Purification Kit (28104) and subjected to Sanger sequencing of the HSP70 gene at the Genomics Sequencing Center at Virginia Tech’s Fralin Life Sciences Institute. The obtained consensus sequences were edited and aligned using Geneious Prime 2022.1.1 software.

To determine the genetic distance model, the PHYML + SMS/OneClick workflow at NGPhylogeny.fr was used (https://ngphylogeny.fr/) (accessed on 20 September 2023) [62]. A Tamura–Nei neighbor-joining tree was constructed using Geneious Tree Builder in Geneious Prime, with bootstrap values estimated from 1000 replicates. This analysis aimed to identify grouping among our samples and match them with the known haplotypes, if possible. We randomly selected and sequenced 40 GLRaV3 isolates from Japan and eight GLRaV3 isolates from Virginia, USA. These isolates were compared with several types of isolates available in the GenBank database.

## 3. Results

### 3.1. Development of the NPN Membrane-Based Grapevine Virus Sampling Kit

#### 3.1.1. Screening for the Type of Membranes, Maceration, and Extraction Methods

Initial testing of previously sampled and frozen grapevine petiole tissue on both FTA cards and NPN membranes using 10 μL spot application was successful for all nine GLRaV1, twenty-five GLRaV2, twenty-five GLRaV3, six GLRaV4, twenty-five GRSPaV, twenty-five GVA, fifteen GVB, six GFkV, eight ToRSV, and twenty-five GRBV positive grapevine samples. Therefore, the FTA card methods proved effective not only for GLRaV1, 2, 3, 5, 9, GVA, GVB, GRSPaV, and GFkV, as previously reported [43], but also for GLRaV4, ToRSV, and GRBV. In addition, the methods previously set forth for Hybond N^+^ Nylon membranes successfully worked to store nucleic acid on NPN membranes using macerated samples. For the rest of the study, we focused on the NPN membrane to explore its capability with grapevine viruses.

Table 3 presents the combinations of macerating buffer, membrane disc treatments, and PCR templates employed to recover GLRaV3 RNA and GRBV DNA. The nucleic acid extraction method using GES with 1% beta-mercaptoethanol as a disc treatment buffer resulted in the best among the tested methods up to a 100% success rate. However, removing the beta-mercaptoethanol from the GES solution resulted in fewer successfully detected positive samples.

Using 2 μL of the supernatant containing the released nucleic acid from the membrane resulted in higher success rates than the blotted 3 mm NPN membrane discs (Table 2). This method achieved a detection rate of 100% for both GLRaV3 and GRBV samples with either EB or GSB. On the other hand, when using the disc template with GLRaV3-positive samples, the detection rate was 83% for EB and 88% for GSB, i.e., we could not detect 17% and 12% of the positive samples, respectively. For GRBV-positive samples, the disc template detection rate was 90% for EB and 94% for GSB.

EB and GSB showed similar success rates in nucleic acid recovery; however, our study chose GSB as the maceration buffer due to its better stability at room temperature. In contrast, untreated and FTA reagent-treated NPN membrane discs showed no success in nucleic acid recovery for GLRaV3 and GRBV, regardless of the maceration buffer or template used (Table 2). Adding Triton X-100 as a washing buffer yielded success rates of up to 50% depending on the combination of maceration buffer and virus being tested.

#### 3.1.2. Virus Detection after an 18-Month Storage

Following the 18-month storage period, 25 samples each of GLRaV2, GLRaV3, GRSPaV, GVA, GVB, or GRBV were detected by RT-PCR or PCR. All samples were successfully amplified, indicating that the NPN membranes retained recoverable amounts of nucleic acid in grapevine sap even after 18 months at room temperature.

### 3.2. The NPN Membrane Kit in the Real World

Survey Results and Genetic Diversity of GLRaV3 among Japanese Isolates

**Figure 1:**
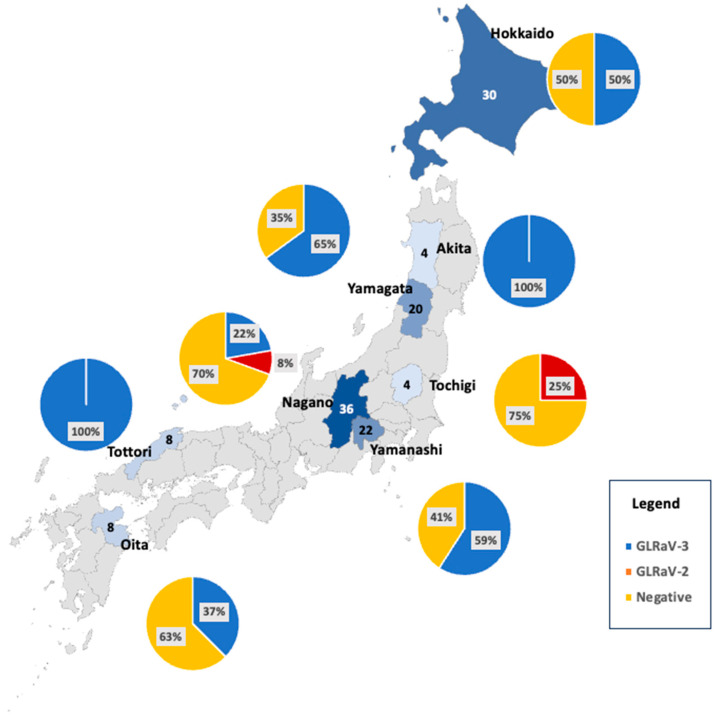
Distribution of GLRaV2 and GLRaV3 among sampled prefectures. The total number of samples per prefecture is shown as a number within the prefecture border, and a darker color indicates more samples per prefecture. Pie charts represent the percentage of virus-positive and negative samples: blue = GLRaV3, red = GLRaV2, and light orange = negative.

A total of 132 independent vineyard block samples were collected from 42 vineyards and nursery operations across eight prefectures in Japan (Figure 1). Among 132 samples, 98 were from the mail-in survey (i.e., 49 out of 50 kits were returned from the growers). These samples represented 29 different cultivars. GLRaV2 detection ranged from 0% to 25% per prefecture (Figure 1), and this virus was found only in two prefectures. The prevalence of GLRaV3 ranged from 0% to 100%, indicating a wide variation in the distribution of this virus among the sampled vineyards. The average across all prefectures was 48.5%. GRBV was not detected in our samples. The negative category representing the absence of GLRaV2, GLRaV3, and GRBV varied across prefectures, ranging from 0% to 75%.

**Figure 2:**
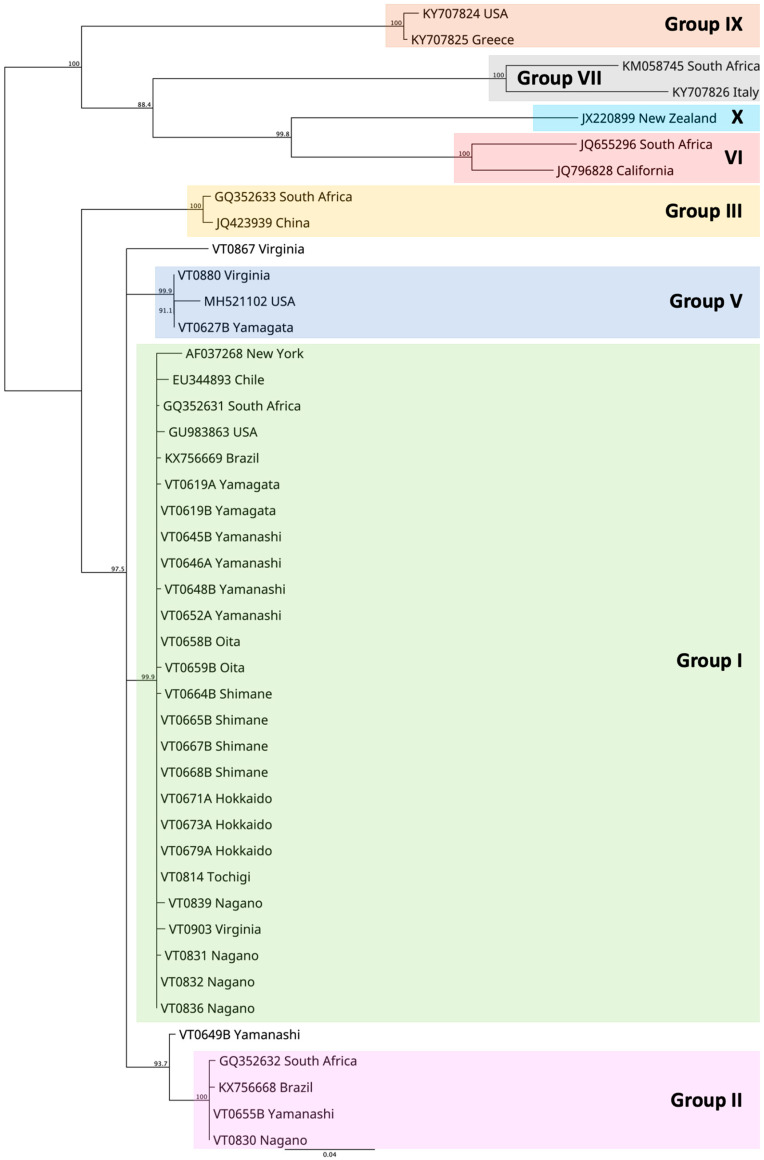
Grouping of GLRaV3 isolates in this study based on the Tamura–Nei neighbor-joining tree with bootstrap values estimated from 1000 replicates (bootstrap values < 80% are not shown).

The phylogenetic analysis revealed that the GLRaV3 isolates from Japan and Virginia clustered within established groups, with two exceptions (VT0867 and VTo629B, Figure 2). Group I encompassed isolates from seven prefectures: Hokkaido, Nagano, Oita, Shimane, Tochigi, Yamagata, and Yamanashi. Group II included isolates from Nagano and Yamanashi, whereas group V comprised Yamagata isolates. VT0649B from Yamanashi prefecture showed a close relation to group II, but further clarification is needed to determine its exact placement within or divergence from group II. Similarly, VT0867 from Virginia appeared closely related to groups I, II, and V, but its precise relationship among these groups remains uncertain.

## 4. Discussion

The first objective of this study was to develop a simple and reliable grapevine tissue sampling tool for grapevine virus detection that vineyard managers could easily implement. This tool, the NPN membrane grapevine virus sampling kit, can efficiently store grapevine sap for subsequent molecular diagnostic assays. We detected various grapevine viruses belonging to families *Betaflexiviridae*, *Comoviridae*, *Closteroviridae*, *Flexiviridae*, *Geminiviridae*, and *Tymoviridae* from samples blotted to the NPN membrane. To the best of our knowledge, this study is the first to use the NPN membrane as a means of storage for grapevine sap for diagnostics of grapevine viruses.

The findings of this study confirmed that viral nucleic acids in grapevine sap can be successfully recovered from the NPN membrane even after a storage period of up to 18 months from the time of sampling under 20–25 °C conditions. Furthermore, these recovered nucleic acids were shown to be amplifiable via conventional PCR, RT-PCR, or qPCR methods.

Previous studies conducted by Chang et al. (2011) and Osman and Rowhani (2006) demonstrated that a membrane blotted with sap can be directly placed in PCR reaction mixtures and tubes for amplification [48,49]. However, this current study’s findings indicated that amplification’s efficacy was lower (88%) even with the best combination of maceration and washing buffers (GES and beta-mercaptoethanol). On the other hand, when the supernatant from the vortexed mix was used as the PCR template, 100% of the samples were amplified. This improved detection rate is likely attributed to the enhanced dispersal of virus nucleic acid particles from the membrane into the solution during vigorous mixing, leading to better amplification and detection in the PCR reactions.

Sample preparation of grapevines for molecular testing can present challenges due to inhibitory phenolic compounds and polysaccharides that impede PCR reactions [43]. These inhibitors, inherent to woody plants such as grapevines, likely account for the suboptimal outcomes observed when water, FTA reagent, or Triton X-100 was used in the reaction mix. Conversely, grapevine-specific buffers formulated to mitigate inhibitory effects have yielded better efficacy in recovering both GLRaV3 RNA and GRBV DNA from the grape sap blotted on the NPN membrane.

It was previously found that FTA cards and Hybond N^+^ Nylon membranes could be used to detect 13 different grapevine viruses (including GLRaV1, 2, 3, 5, 9, GVA, GVB, GRSPaV, GFkV, GFLV, ArMV, prunus necrotic ringspot virus (PNRSV), prune dwarf virus (PDV)), *Xylella fastidiosa*, and multiple phytoplasma nucleic acids from the blotted grape sap [48,49]. This study confirmed that the FTA card method was also successful with GLRaV4, ToRSV, and GRBV. However, one of the drawbacks of the FTA card was that the sample could last only for up to four days to 2+ months [49]. This was another reason for focusing on the NPN membrane in this study.

This kit offers several immediate and short-term advantages. First, it simplifies the transport of membranes to and from the research laboratory, removing the need for coolers or costly express services. Second, the stability of nucleic acid on the NPN membrane for up to 18 months at room temperature (20–25 °C) eliminates the requirement for cold storage. Looking towards the long-term benefits, we envision the application of this kit (with more studies) for the storage and testing of virus-, bacteria-, or phytoplasma-infected samples from grapevines or other crops. The kit can be a great tool for collecting samples in remote locations lacking access to cold storage facilities or expedited shipping services.

The second objective of this study involved surveying GLRaV2, GLRaV3, and GRBV viruses within Japanese vineyards. The NPN membrane grapevine virus kit was particularly advantageous during the global COVID-19 pandemic. Despite the physical distance between the Principal Investigator in the USA and the vineyard in Japan, the kit and detailed instructions enabled sample collection. Thus, this survey became a realistic validation study of the use of the kit. We distributed the kit and detailed instructions (in writing and as a video). We also targeted vineyard managers, vineyard or winery owners, and nursery owners keen on the issue. A 98% return rate for the kit showed that this kit could be helpful, especially if we identified vital people in the group. To our knowledge, this is the first example of the actual use of these types of kits by growers.

The survey revealed that 48.5% of Japanese vineyards and nursery blocks were infected with GLRaV3. When summed for the operation level, 62% of operations (42 total) resulted in at least one vineyard block sample being positive with GLRaV3. In a previous study conducted in Virginia, GLRaV3-positive vines were found in 25% of 100+ samples, and at least one of nine tested viruses was present in 64% of the surveyed vineyards [18]. A study in 2009 in the Finger Lakes region of New York found that GLRaV1, GLRaV2, or GLRaV3 were detected in 68% of the sampled vineyard blocks [11], but GLRaV3-positive vines were found only in 10% of their sampled vineyard blocks. Although the sample size is limited, our data suggest that GLRaV3 is widely distributed in Japanese vineyards compared with other surveyed countries.

GLRaV2 was found in vineyards located in Nagano and Tochigi prefectures. Since there is no known vector of GLRaV2, propagation is considered the main route for the spread of GLRaV2 [63]. Further investigation is warranted since it was found in three different cultivars (Norton, Muscat Bailey A, and Pinot gris) across four geographically distant vineyards.

GRBV was not detected in the samples we obtained. While this does not guarantee the absence of GRBV, the risk of its presence appears to be low in Japan, likely due to the limited importation of grapevines until recent times. In contrast, reports from various countries such as Canada, India, Italy, France, Korea, Mexico, and Switzerland [27,32,64,65,66,67,68,69] indicate that infected materials have been distributed worldwide.

The higher incidence of GLRaV3 can be attributed, in part, to the absence of a certified vine program in Japan. Also, we learned that it is common for nurseries to obtain scion budwoods from commercial vineyards instead of having their own source (Nita, personal communication). Some major nurseries are in Yamagata prefecture, where 65% of samples were GLRaV3 positive. Samples from Yamagata prefecture also contained two GLRaV3 haplotypes. The only other prefecture with two haplotypes was Nagano. However, we received relatively more samples from these two prefectures, and sample sizes from other prefectures were too small to make a solid conclusion.

Among the Japanese GLRaV3 isolates, we identified three haplotypes. Although only one gene was used in this study and further analyses are required to obtain better resolution, the result suggested the possibility of multiple introductions of GLRaV3-infected plant materials into Japan. However, the lack of proper documentation and the regular exchange of plant materials between nurseries and growers have made it challenging to trace the origin of these materials at present. Thus, our research group is collaborating with the Japan Vineyard Association to investigate the movements of the scion budwoods exchange among growers and nurseries. We also initiated an importation and quarantine program for cleaner nursery materials in 2019.

## 5. Conclusions

We have successfully developed a straightforward and reliable NPN membrane-based method for sampling and storing viruses in grapevine sap. We devised an efficient extraction method of nucleic acid from the NPN membrane, resulting in 100% amplification success for GLRaV2, GLRaV3, GRSPaV, GVA, and GVB in RT-PCR, and GRBV in PCR. This method enables growers to blot grapevine sap onto the membrane, allowing for the storage of recoverable nucleic acid for a minimum of 18 months at a temperature range of 20 to 25 °C. The sampling kit was used for a virus survey of Japanese vineyards, which revealed a substantial prevalence of GLRaV3.

## Figures and Tables

**Table 1:** Primers used for PCR and RT-PCR for the detection of grapevine viruses in the membrane study.

Virus	Primer Name and Reference	PCR Parameters (Temp in °C)	Number of Samples Tested ^a^
GLRaV1	dCP1-1/dCP1-2 [54]	52°, 1 h; 35 x (94, 30 s; 54, 45 s; 72, 1 min); 72, 2 min	9
GLRaV2	P19qtF4 and p24qtR [55]	52°, 1 h; 35 x (94, 30 s; 54, 45 s; 72, 1 min); 72, 2 min	25
GLRaV3	GEN-11112F/GEN-11233R [56]	52°, 1 h; 35 x (94, 30 s; 54, 45 s; 72, 1 min); 72, 2 min	25
GLRaV4	LRAmp-F/LRAmp-R [25]	52°, 1 h; 94, 2 min; 40 x (94, 30 s; 50, 35 s; 72, 45 s); 72, 7 min	6
GRSPaV	RSP13/RSP14 [57]	52°, 1 h; 35 x (94, 30 s; 54, 45 s; 72, 1 min); 72, 2 min	25
GFkV	GFkV-585 F/GFkV-1117 R [37]	52°, 1 h; 35 x (94, 30 s; 54, 45 s; 72, 1 min); 72, 2 min	6
GVA	H587/C995 [58]	52°, 1 h; 35 x (94, 30 s; 54, 45 s; 72, 1 min); 72, 2 min	25
GVB	C410/H28 [58]	52°, 1 h; 35 x (94, 30 s; 54, 45 s; 72, 1 min); 72, 2 min	15
ToRSV	ToRSV5/ToRSV6 [59]	50°, 30 min; 94, 2 min; 30 x (94, 45 s; 60, 45 s; 68, 2 min); 68, 5 min	8
GRBV	GVGF1/GVGR1 [28]	94°, 2 min; 35 x (94, 30 s; 60, 30 s; 72, 1 min); 72, 5 min	25

^a^ Samples were collected in our previous studies [6,18].

**Table 2:** GLRaV3 and GRBV nucleic acid recovery rate from NPN membranes.

Maceration Buffer	Method	Washing Buffer ^a^	Template for PCR ^b^	GLRaV3 ^c^	GRBV ^c^
EB	A	No treatment	Disc	0/48	0/48
	B	Triton X-100	Disc	16/48	23/48
	C	FTA reagent	Disc	0/48	0/48
	D1	GES	Disc	33/48	41/48
	D2	GES	2 μL solution	37/48	44/48
	E1	GES + beta-m	Disc	40/48	43/48
	E2	GES + beta-m	2 μL solution	48/48	48/48
GSB	A	No treatment	Disc	0/48	0/48
	B	Triton X-100	Disc	13/48	24/48
	C	FTA reagent	Disc	0/48	0/48
	D1	GES	Disc	31/48	43/48
	D2	GES	2 μL solution	34/48	47/48
	E1	GES + beta-m	Disc	42/48	45/48
	E2	GES + beta-m	2 μL solution	48/48	48/48
Water control	A	No treatment	Disc	0/48	0/48
	B	Triton X-100	Disc	0/48	0/48
	C	FTA reagent	Disc	0/48	0/48
	D1	GES	Disc	0/48	0/48
	D2	GES	2 μL solution	0/48	0/48
	E1	GES + beta-m	Disc	0/48	0/48
	E2	GES + beta-m	2 μL solution	0/48	0/48
Positive control	Traditional nucleic acid extraction method without the membrane [6,18]			48/48	48/48

^a^ beta m = 1% beta-mercaptoethanol. ^b^ Disc = Membrane placed directly in a PCR tube, 2 μL = a tube containing a membrane, and the washing buffer was vortexed for 90 s, and then 2 μL of supernatant was taken from the tube for PCR. ^c^ Number of samples correctly identified as positive/total known positive samples tested.

**Table 3:** The NPN membrane grapevine virus sampling kit and its procedures, Japanese vineyard grapevine survey 2020–2021.

Petiole collection
Arbitrarily select fresh grapevine petiole samples from the vine’s canopy. Sample both young and old leaves from different parts of the canopy.
Record the cultivar and note any observed symptoms at the time of sampling.
(Optional) Store the sample in a freezer overnight to soften the tissue.
Blotting
Cut the petiole samples into small pieces using a disposable razor blade.
Place the petiole cuttings into the bottom of a disposable medicine cup until a layer of tissue covers the bottom.
Add pre-measured GSB (1 mL) to each sample cup.
Use sterile 15 cm long wood applicators with blunt ends to mash the petiole tissue for 60 to 90 s until the color of the buffer becomes green, and then macerate the tissue for another 60 to 90 s.
Dip the wood applicator into the mixture and gently touch/blot it onto an NPN membrane; blot thrice in one spot, and then repeat twice to create three spots per sample.
Allow the membrane to dry for at least 24 h, and then cover the surface with the provided protective paper.
Nucleic acid extraction and PCR
Remove a 3 mm disc of the blotted membrane using a sterile micro-punch.
Place the disc in a 200 μL microcentrifuge tube.
Add 50 μL of GES containing 1% beta-mercaptoethanol to the tube.
Incubate the tubes at 95 °C for 10 min, and then vortex for 90 s.
Place the tubes on ice.
Use 2 μL of the supernatant from the tubes containing discs as a template in a 25 μL total volume PCR reaction specific for each virus using the appropriate primer set (Table 1 and Table 2).

**Table 4:** Primers (denoted as f and r) and probes (denoted as p) used in RT-qPCR, qPCR, and traditional PCR of GLRaV2, GLRaV3, and GRBV for detection and sequencing of Japanese samples collected using the NPN grapevine virus sampling kit.

Virus	Gene	Primers and Probes	Reference
*RT-qPCR*			
GLRaV2	heat shock protein (HSP70)	LR2-124f	[60]
		LR2-284r1	
		LR2-284r2	
		LR2-284r3	
		LR2-214p	
GLRaV3	3′ untranslated terminal region (UTR)	LR3-FPST-F1	[56]
		LR3_FPST-F2	
		LR3_FPST-F3	
		LR3_FPST-F4	
		LR3_FPST-R1	
		LR3_FPST-R2	
		LR3_FPST-P1	
		LR3_FPST-P2	
*qPCR*			
GRBV	V2	RB3-F	[20]
		RB3-R	
		RB3-P	
Plant	COX	COX-F	[61]
		COX-R	
		COX-P	
*RT-PCR for sequencing*			
GLRaV2	heat shock protein (HSP70)	LR2-U2	[39]
		LR2-L2	
GLRaV3	heat shock protein (HSP70)	LC1	[49]
		LC2	
*PCR for sequencing*			
GRBV	V2	GVGF1	[29]
		GVGR1	

## Data Availability

All data, except personal information, are available in tables and figures.
